# Palatal fenestration after orthodontic treatment. Intermodal approach and evolution. A case report

**DOI:** 10.4317/jced.55376

**Published:** 2018-12-01

**Authors:** Pablo Pérez-Lanza, Pedro Almiñana-Pastor, Francisco Alpiste-Illueca

**Affiliations:** 1 Department of Stomatology, Faculty of Medicine and Dentistry, University of Valencia (Spain). C/ Gascó Oliag 1, 46010 Valencia Spain

## Abstract

A 15-year-old patient is referred to the Department of Periodontics of the University of Valencia. The patient reported dissatisfaction with the position of upper right canine after orthodontic treatment. Previously, in a private center, surgical approaches were performed for the traction of the canine included. On clinical examination at first appointment, generalized plaque-induced gingivitis was observed, with palatal fenestration of the root of upper right lateral incisor of approximately 75% of the total surface, with suppuration and very little gingiva inserted at the cervical level, which presents a buccal torque of the crown. Radiologically, a mild rhizolysis and bone loss adjacent to upper right lateral incisor was observed. An interdisciplinary treatment is decided between the departments of Orthodontics, Endodontics and Periodontics: - Canal treatment of upper right lateral incisor, performing a retrograde filling of the cavity with Biodentine ® (Septodent, Saint Maur de Fossés, France). - Orthodontic treatment, modifying the torque and improving the stability. - Periodontal treatment, performing a connective tissue graft by Langer technique adapted to the case. After the conclusion of the orthodontic treatment, an improvement in the situation is observed. The graft was performed, presenting at 3 months a line of fenestration in the mucosa. At 4 years, the patient is asymptomatic, without suppuration, with a total closure of the fenestration.

** Key words:**Recession, orthodontics, connective tissue graft.

## Introduction

Gingival recessions after orthodontic treatment have been widely treated in the scientific literature, considering that for it to occur the tooth must be moved out of the alveolar bone. That is, the presence of a bone dehiscence is an essential requirement for the presence of a recession (1). Classic experimental studies indicate that, after repositioning the tooth in a correct root position, it is possible that a new bone formation occurs in the area of dehiscence (2). It has been reported that the presence of inflammation and thin gingival tissue, which can be produced by the stretching of the gingival tissues during orthodontics, are important predictors of gingival recession (3). The following is a clinical case in which, as a result of an inadequate orthodontic treatment linked to the traction of an impacted canine, a recession in the palatal tissue has occurred.

## Case Report

15 year old patient, with no medical history of interest, who attended the Department of Periodontics of the University of Valencia, sent from a private center. In this center, she expressed her disagreement with the aesthetic result of the orthodontic treatment to which the patient was submitting at that time (May 2013), due to the buccal and coronal position of the upper right canine. After the examination, a generalized plaque-related gingivitis was diagnosed and a palatine fenestration of the root of the upper right lateral incisor.

**Figure 1:**
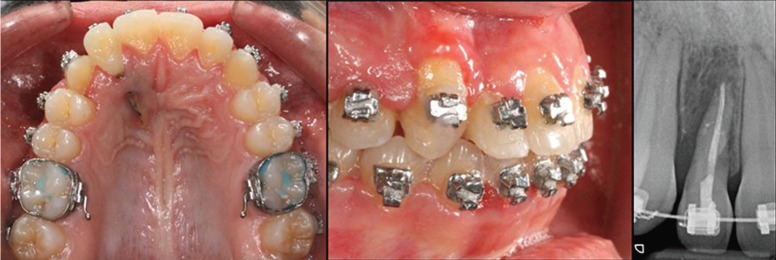
Initial situation. Plaque-related gingivitis and the fenestration of the palatal tissues are observed.

Already in the Periodontics Department, the patient reported that the upper right canine was pulled through orthodontics after performing two approaches (palatal and vestibular). In the clinical examination, previous diagnoses are confirmed and suppuration to the palpation is observed in the palatal fenestration, which has an extension of approximately 75% of the total surface of the root of upper right lateral incisor. This lateral incisor presents, as a consequence of orthodontic treatment, an accentuated buccal torque of the crown. As for the upper right canine, a band of gum inserted narrowly at the cervical level was observed. On radiological examination, a rhizolysis of the apical third of the root of the upper right lateral incisor was observed, due to the traction of the adjacent tooth and loss of interproximal bone (Fig. 1).

At that time, the patient was informed of an unfavorable prognosis (4) for that tooth, as well as the different treatment options. The removal and placement of an implant is discouraged due to the age of the patient with the consequent complications that may occur in the medium term (5). An interdisciplinary treatment plan was elaborated:

- Endodontics department; upper right lateral incisor root canal treatment and retrograde filling with Biodentine®

- Orthodontic department; occlusal stabilization and correction of the position of the upper right lateral incisor and upper right canine.

- Periodontics department; basic periodontal phase and treatment of palatal fenestration using the Langer technique (6) adapted to the case.

After the completion of the endodontics in November 2013 and with the basic periodontal phase and oral hygiene instructions concluded, an orthodontic treatment was initiated, which ended one year later. Initially, after performing a CBCT, it was determined to perform a palatal graft with obturation of root resorption with Biodentine ® (7). After orthodontics, a significant reduction in palatal root exposure was observed.

**Figure 2:**
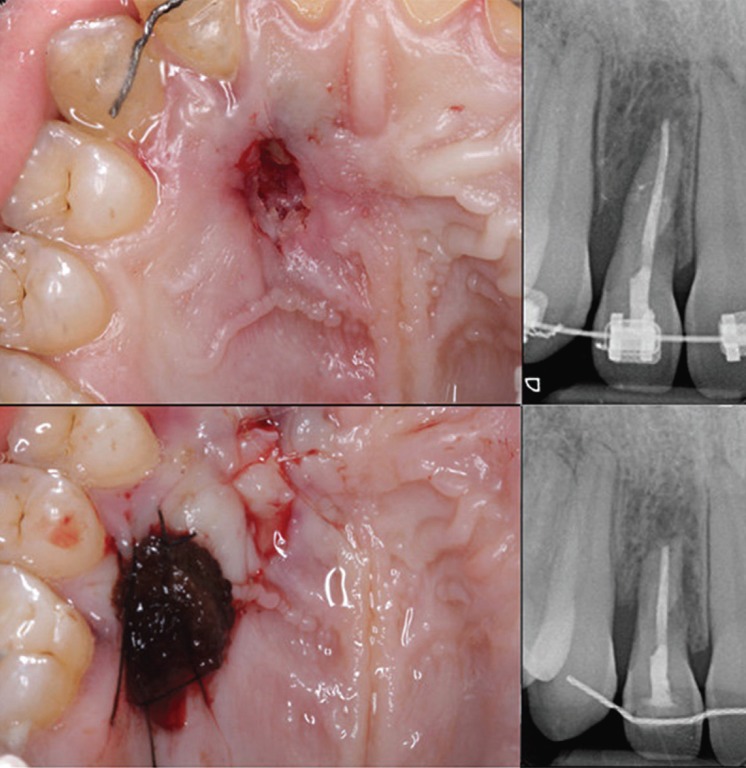
Surgical procedure consisting on the apicoectomy and the connective tissue graft.

In the surgery, the Langer technique (6) was modified to adapt it to the case. A palatal incision was made, with the excision of the edge of the fenestration for histological analysis. A partial thickness flap was raised in the receiving area. In the donor area, a full-thickness graft was harvested, placing Surgicel ® (Ethicon, Johnson & Johnson Medical Devices & Diagnostic Group, Miami, FL, USA) in the wound, suturing it with Supramid ® 4/0. Although the obturation was initially considered with Biodentine ®, due to the apical destruction, an apicoectomy was performed with retrograde filling. It was sutured with resorbable 5/0 of polyglycolic acid suture in the recipient bed and Supramid ® (SMI, St.Vith, Belgium) 4/0 in the donor area. Amoxicillin 500 mg and anti-inflammatory therapy was prescribed (Fig. 2)

**Figure 3:**
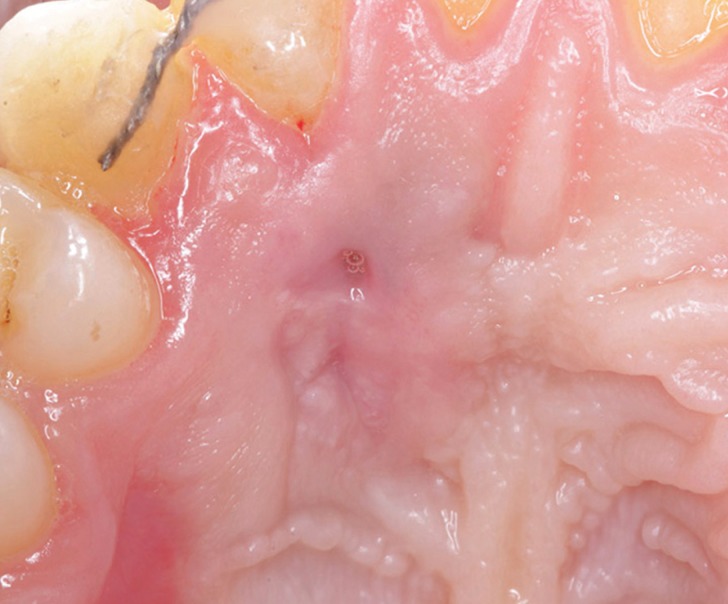
3-year evolution of the palatal fenestration.

One month later, a small remnant fenestration was observed, approximately one millimeter in diameter. The patient was maintained at periodic recalls every 3 months initially and, once the stability had been checked, every 6 months. 3 years later the tooth is asymptomatic and without suppuration, with closed fenestration (Fig. 3). There is a lack of vestibular volume at level of the upper right lateral incisor. Despite the scarce gum inserted at the upper right canine level at the initial time, the patient does not present mucogingival problems.

## Discussion

In this case a series of complications related to orthodontic treatment for the traction of an impacted canine are addressed. The presence of these included teeth is estimated at 2% of the population, with a prevalence of 2:1 in women and 2:1 in maxilla (8). Within them, approximately 66% are palatinized (9). The etiology is uncertain, although the discrepancy of the length of the arch seems to be a primary etiological factor (10).

From a periodontal point of view, several studies confirm that the traction of an included canine is not a risk, although it is influenced by the initial position of the canine both horizontally and vertically (11). In this case, two complications derived from the treatment are observed; on the one hand, the scarce gingiva inserted in cervical of upper right canine and, on the other hand, the root fenestration of the upper right lateral incisor.

For a soft tissue recession to occur there are several factors that can happen simultaneously. First, the root of the affected tooth must be positioned outside the alveolar process (1). However, this does not ensure the recession since, in thick biotypes, although there has been a loss of bone tissue, it is possible that the gum remains in the same position. In any case, an inflammatory stimulus can trigger the onset of recession, with its area of involvement being especially significant for thin biotypes (12).

Regarding the fenestration of the upper right lateral incisor and, based on the comments made, it can be speculated that the excessive buccal torque of the crown and, therefore, palatal of the root, has propitiated a position of the root outside the palatal cortex. In addition, the hypothesis is established that the affectation of the apical third of the root has triggered an inflammatory stimulus that, in addition of the reduced thickness of the palatal mucosa because of the torque of the root, has favored this fenestration. We opted for orthodontic treatment prior to surgery to move the root away from the mucosal surface, so that the closure of the tissues was more favorable.

Likewise, the upper lateral incisor presents a rhizolysis due to traction of the canine. It is a phenomenon with variability in terms of incidence between 3% (13) and 33% (14).

On the other hand, upper right canine presents an almost total absence of inserted gingiva. Despite the initial controversy in the literature, it is accepted that a band of gum inserted very narrow or null is compatible with the maintenance of periodontal health (15). Because the patient keeps a correct hygiene, a conservative approach was chosen.

The sum of variables gives the tooth a questionable prognosis (4), since after the periodontal treatments survival and stability can not be guaranteed. However, several factors caused that the

option chosen was the conservation of the piece; the age of the patient, taking into account the changes that occur in the peri-implant tissue in adolescents (5) and the complications that arise in the implants, with only 39.3% of the implants free of complications at 9 years (16) .

Therefore, once the endodontic and orthodontic treatments were completed, the apicoectomy was carried out and the connective tissue graft from the adjacent bed was placed. The results at 3 years are favorable, having achieved a total closure of the fenestration and maintaining stability and correct periodontal health.
